# Bioengineering Innovations for Personalized Care in Low Back Pain: From Sensors to Smart Therapeutics

**DOI:** 10.3390/bioengineering13020212

**Published:** 2026-02-12

**Authors:** Jiri Gallo, Michal Stefancik, Petr Mik, Lenka Lhotska

**Affiliations:** 1 Department of Orthopedics, Faculty of Medicine and Dentistry, Palacky University, University Hospital, 779 00 Olomouc, Czech Republic; michal.stefancik@fnol.cz (M.S.); petr.mik@fnol.cz (P.M.); 2 Department of Cognitive Systems and Neurosciences, Czech Institute of Informatics, Robotics, and Cybernetics, 160 00 Prague, Czech Republic; lenka.lhotska@cvut.cz

**Keywords:** low back pain, heterogeneity, biosensors, polyEMG, pain analysis, biomarkers, wearable technology, adaptive rehabilitation, precision medicine

## Abstract

Low back pain (LBP) remains one of the most prevalent and disabling musculoskeletal conditions worldwide, shaped by interacting mechanical, neurophysiological, inflammatory, vascular, and behavioral factors. Conventional care often relies on generalized exercise programs and episodic, predominantly subjective assessment, which can underrepresent inter-individual heterogeneity and longitudinal change. Recent bioengineering advances enable continuous, multimodal monitoring of objective correlates of function—neuromuscular activation and coordination (sEMG/polyEMG), movement patterns and activity exposure (IMU), and complementary physiological context (e.g., autonomic and perfusion-related signals). Rather than measuring pain directly, these signals can contextualize symptoms, support treatment stratification within non-surgical care, and enable trajectory monitoring with early non-response flags to guide timely rehabilitation adjustment under clinician oversight. When integrated with transparent, implementation-oriented analytics, biosensing can also support incremental closed-loop rehabilitation through patient-facing feedback and adaptive progression rules. This review synthesizes current and emerging biosensing approaches for LBP and highlights key translational requirements—outcome-linked validation, standardization, and workflow integration—to bridge engineering innovation with clinically usable, data-informed rehabilitation.

## 1. Introduction

Low back pain (LBP) is a multifactorial syndrome characterized by lumbosacral pain with variable stiffness, movement limitation, altered muscle activation, and—in some presentations—neurogenic symptoms [1]. Symptoms often fluctuate with posture and activity exposure, and LBP remains among the leading causes of disability worldwide with substantial societal and healthcare burden [2].

Clinically, LBP is often categorized as “specific” (identifiable structural, inflammatory, or neurological cause) or “non-specific,” yet this dichotomy is increasingly insufficient [3]. Many patients exhibit co-existing structural and functional abnormalities—degenerative findings, altered lumbopelvic coordination, protective co-contraction, reduced movement variability, or fatigue-related deterioration—none of which is universally causal in isolation (Table 1). The practical challenge is therefore not the absence of abnormalities, but objective characterization of how an individual moves, loads, and adapts over time, and how functional patterns relate to symptoms, activity tolerance, and response to rehabilitation [4,5].

**Figure 1:**
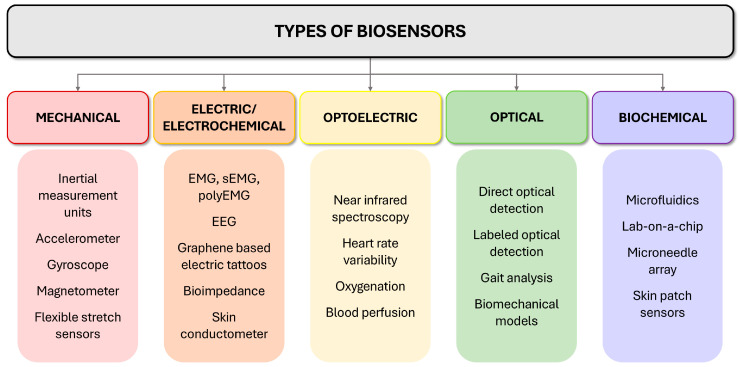
Overview of major biosensor classes relevant to musculoskeletal and LBP research. Schematic taxonomy grouping commonly used sensing approaches into mechanical, electrical/electrochemical, optoelectronic, optical, and biochemical modalities, with representative examples listed under each category (e.g., IMUs; EMG/EEG and epidermal electronics; near-infrared/oxygenation and perfusion proxies; optical detection and gait/motion analysis; and microfluidic/lab-on-a-chip sampling platforms). Categories are intended as a practical overview; in real-world systems, multimodal devices and data-fusion pipelines often span more than one class.

This demand for greater precision has accelerated the integration of bioengineering tools that capture objective, continuous, and patient-specific data [7,8]. Wearable and surface biosensors—implemented via inertial measurement units (IMUs), (multi-channel) surface electromyography (sEMG), smart textiles, and flexible sensing platforms—enable high-resolution monitoring of musculoskeletal and physiological signals and can quantify movement and muscle activation while characterizing coordination strategies and compensatory behavior. Key types of sensors and modalities, outputs, and limitations are summarized in Figure 1 and Table 2.

Beyond measurement, biosensors can support adaptive rehabilitation by enabling feedback and longitudinal tracking that inform individualized progression of exercise and lifestyle strategies. This review synthesizes current and emerging biosensor applications for LBP and outlines an implementation-oriented pathway from sensing to clinically actionable, personalized rehabilitation.

## 2. The Role of Biosensors: From Measurement to Meaning

To meaningfully advance clinical management of LBP, multimodal sensing must move beyond passive data capture toward clinically interpretable, reproducible measures that support individualized rehabilitation. In contrast to single time-point clinical assessments, biosensors can quantify how a patient moves and loads over time—capturing kinematics, muscle activation strategies, and activity exposure in daily-life contexts [20,21,22,23].

Importantly, biosensors do not diagnose pain mechanisms or identify a single anatomical pain generator in isolation [6]. Rather, IMU–EMG (and related) signals provide objective correlates of functional behavior, e.g., altered lumbopelvic coordination, protective co-contraction/guarding, asymmetry, reduced variability, and fatigue-related deterioration that can support clinical phenotyping and guide mechanism-informed decision-making when integrated with history, examination, imaging where indicated, and patient-reported outcomes [20,24,25,26,27]. In this sense, sensing helps operationalize “movement quality” and “stability-related behavior” in a way that is trackable and comparable across time.

Multimodal systems integrating IMUs and sEMG (Table 2), optionally complemented by pressure or physiological sensing, enable (i) task-specific quantification of spinal and lumbopelvic motion, (ii) characterization of muscle recruitment, site asymmetries and coordination strategies, and (iii) longitudinal monitoring of change during rehabilitation [28,29]. This longitudinal perspective is clinically relevant because dysfunctional patterns often emerge from intersegmental dependencies across the pelvis–spine–lower-limb chain rather than from an isolated impairment.

**Figure 2:**
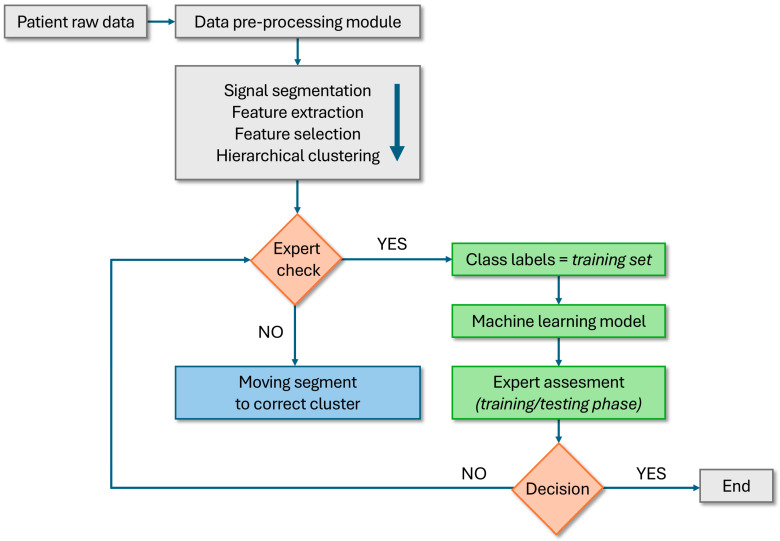
Expert-in-the-loop semi-automatic classification of IMU–EMG patterns. Multimodal raw signals (IMU and sEMG) are preprocessed (filtering; EMG rectification/envelope; time-frequency features where applicable), then transformed into features, reduced/selected, and grouped using hierarchical clustering. An expert reviews cluster assignments and, when needed, corrects labels by reassigning a representative epoch closest to the cluster centroid; the full cluster is then relabeled automatically. This semi-supervised workflow supports training-set development and enables robust handling of previously unseen patterns. The arrows show the direction of the process, the flow of signals and data in the system.

Beyond measurement, wearable sensing can support self-management through feedback and adherence monitoring. Real-time cues (e.g., posture reminders, movement-quality targets, muscle engagement feedback) and app-based coaching can reinforce motor retraining and facilitate sustained behavior change [30,31,32,33]. When coupled with robust preprocessing, transparent reporting, and clinician oversight—particularly for atypical or previously unseen patterns—biosensing can enable adaptive progression of exercise and lifestyle strategies within a continuous, data-informed care pathway (Figure 2). Section 3.1, Section 3.2, Section 3.3, Section 3.4 and Section 3.5 summarize the main sensing layers, while their necessity and reasons for clinical use are listed in Table 3.

## 3. Bioengineering Sensors in LBP Management

### 3.1. Wearable Sensors, Smart Textiles, and Graphene-Based Devices

Wearable technologies are increasingly used for functional assessment and longitudinal monitoring in LBP [24,30,34,35]. For clinical translation, wearables must be skin-compatible and robust over extended use, provide stable signal quality under real-world conditions, and remain scalable in cost and manufacturing. These constraints often determine translational success as much as the sensing principle itself.

Inertial sensors (accelerometers, gyroscopes, magnetometers; IMUs) can be integrated into garments or adhered to the skin to track posture, lumbar and lumbopelvic kinematics, gait proxies, and activity exposure in daily life (Table 2). Rather than “diagnosing” a source of pain, these measures help quantify functional patterns relevant to rehabilitation, e.g., altered coordination, asymmetry, reduced movement variability, or fatigue-related deterioration, especially when interpreted alongside clinical assessment and patient-reported outcomes. Signal quality metrics and placement checks are essential to avoid false “phenotypes” driven by artefacts.

Smart textiles extend this approach by embedding conductive fibers and flexible sensor arrays into clothing, supporting comfortable, low-burden monitoring suitable for home use [35,36,37,38]. Multimodal fusion (e.g., IMU with sEMG and, where relevant, pressure/force sensing) can enrich interpretation by linking how the body moves with how it is controlled, enabling a more complete description of movement strategy and compensation during daily tasks [39]. However, signal quality, placement stability, and data synchronization remain key practical limitations that must be reported transparently.

Graphene-based electronic tattoos and other ultrathin epidermal electronics represent an emerging direction for unobtrusive, high-fidelity sensing [40,41]. Their main promise lies in conformal skin contact and improved comfort during prolonged monitoring, potentially enabling higher-quality electrophysiological and physiological recordings in mobile settings. For LBP, these platforms remain largely pre-translational; their near-term value is best framed as enabling technology requiring further validation and workflow integration.

### 3.2. Devices for Analyzing the Muscular Component of Low Back Pain

Spinal posture and stability reflect interactions between passive structures and active muscular control. Imaging (ultrasound, MRI, CT) can reveal structural muscle changes (atrophy, fatty infiltration, asymmetry) associated with chronic LBP, but it does not capture real-time function during movement or task exposure [42].

**Figure 3:**
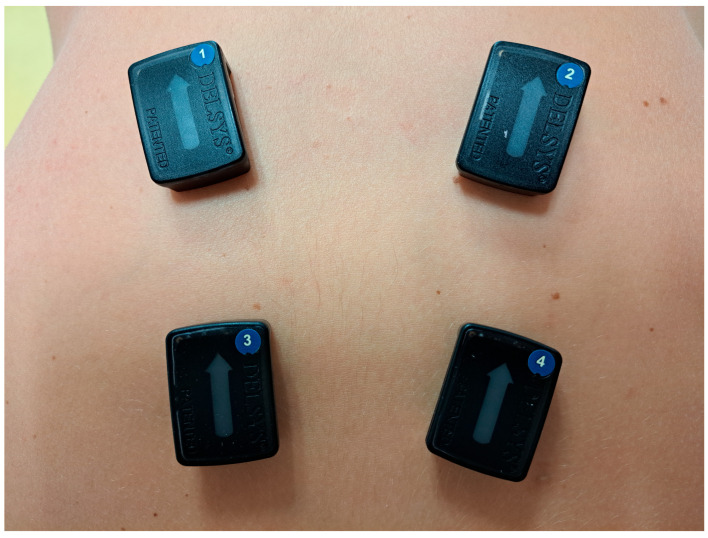
Example of multi-channel EMG (polyEMG) electrode placement on the lower back. Illustrative placement of four bipolar electrode pairs along the lumbar region to capture spatial differences in activation across segments. Multi-channel EMG can be synchronized with kinematic sensing (e.g., IMU) to relate muscle activation strategies to movement execution during functional tasks and rehabilitation. In our feasibility experiments, EMG was acquired using commercially available wireless systems (Shimmer and Delsys); the focus was on long-duration recording and the acquisition/analysis pipeline to assess signal quality and practical feasibility over extended monitoring, rather than on developing new hardware.

**Figure 4:**
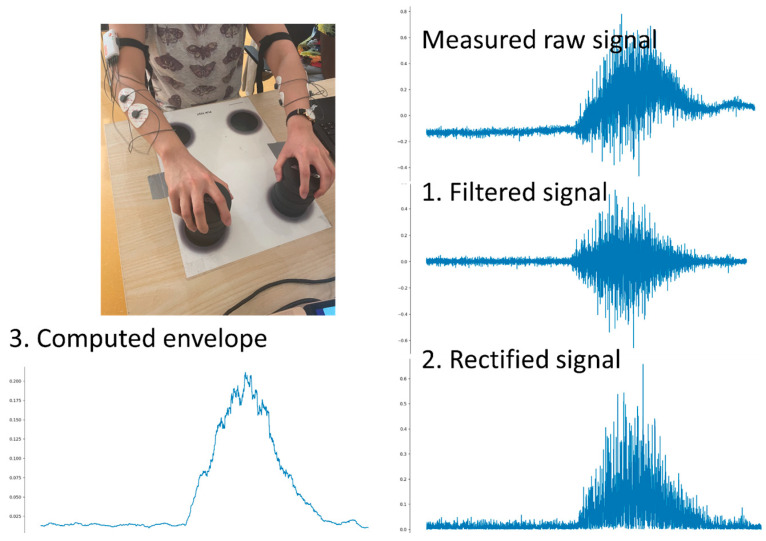
Example of an experimental recording setup. A wearable surface EMG system enables recording during natural movement. In our feasibility experiments, raw EMG–IMU streams were acquired via manufacturer software (with time-stamping/synchronization) and stored locally for offline preprocessing (Butterworth filtering, rectification, envelope and time–frequency features), with quality control supported by an expert-in-the-loop review of atypical segments. EMG workflows typically include filtering, envelope computation, and feature extraction (e.g., amplitude/timing metrics and, where relevant, frequency-domain fatigue proxies), with transparent reporting of sampling, synchronization, and artifact handling. Combined with IMU, these systems support longitudinal monitoring and individualized rehabilitation progression.

Surface EMG offers dynamic insight into neuromuscular behavior, and multi-site/multi-channel approaches (polyEMG) (Figure 3 and Figure 4) extend conventional recordings by sampling multiple regions or muscle groups simultaneously [43,44,45]. In LBP research, such recordings have been used to characterize activation strategies, timing relationships, asymmetry, fatigue signatures, and guarding/co-contraction patterns during static postures and functional tasks [46,47]. Importantly, these signals reflect motor behavior under pain and load and should be interpreted as functional correlates rather than direct indicators of a single pathology.

When synchronized with kinematic sensing (e.g., IMU), multi-channel EMG can help link electrical activation to movement execution and reveal task-specific coordination strategies across the lumbopelvic chain. This supports individualized rehabilitation planning (e.g., targeting excessive co-contraction, improving timing/control, addressing endurance deficits) and allows objective tracking of change over time [22,48,49]. At the same time, the clinical evidence base for polyEMG in routine LBP care remains limited; studies are often small and methodologically heterogeneous, with variable protocols and outcomes. Standardized acquisition, reporting, and interpretation frameworks—together with outcome-linked validation—are therefore essential before broad clinical adoption.

Building on prior EMG and LBP rehabilitation work [50,51], long-duration surface EMG monitoring has emerged as a promising—yet methodologically demanding—approach for capturing real-world neuromuscular behavior beyond short laboratory tasks. In this line of work, the focus is on processing continuous, hours-long EMG recordings with synchronized auxiliary signals, and on incorporating an expert-in-the-loop step to improve training-set curation and model refinement when novel patterns arise (Figure 2) [52]. Such frameworks can support individualized rehabilitation by integrating patient-specific baselines with longitudinal change during therapy, under clinician oversight.

### 3.3. Toward Biosensing of Pain

Pain is inherently subjective and remains best captured through patient self-report using validated instruments [6]. In LBP, symptom intensity and interference can vary substantially across individuals with similar imaging findings, and pain mechanisms may be mixed and dynamic over time. These features limit any single “objective” pain measure and complicate outcome evaluation.

Current biosensors do not measure pain directly. Their value is in capturing correlates of pain-related physiology and behavior, which can complement self-report and clinical assessment [20,53,54,55,56]. Relevant domains include (i) neuromuscular patterns (e.g., guarding/co-contraction, altered timing, fatigue-related changes), (ii) autonomic arousal and recovery proxies (e.g., heart rate variability (HRV), electrodermal activity, temperature), (iii) brain-related signals in research contexts (e.g., electroencephalography (EEG) or other neuro-sensing modalities), and (iv) movement behavior and activity exposure captured via kinematics. These signals can help contextualize fluctuations in symptoms, identify task sensitivity, and track rehabilitation-relevant functional change without implying that a sensor can “verify” pain or replace the patient’s report [5,57].

A pragmatic translational direction is triangulation: combining repeated self-report (intensity, interference, function) with objective sensing during standardized tasks and in daily life, to model within-person change and treatment response [1,2,49]. This approach prioritizes longitudinal patterns, reproducibility, and clinically interpretable endpoints rather than attempting to infer a single pain generator or definitive pain “type” from biosignals alone.

### 3.4. Monitoring of Inflammatory and Perfusion Biomarkers

LBP is not solely biomechanical; inflammatory signaling, metabolic stress, and microcirculatory factors may contribute to symptom persistence in subgroups of patients [58,59]. Accordingly, physiological biosensing may complement neuromuscular and kinematic monitoring by providing context on systemic or local stress responses and recovery.

Low-grade inflammation has been associated with chronic pain states and may influence nociceptor sensitivity and tissue repair [4,60,61]. Emerging wearable biochemical sensors (e.g., sweat or interstitial-fluid sampling platforms) aim to detect metabolites, stress-related hormones, or inflammatory mediators, but most approaches remain early in validation and require careful interpretation due to biological variability and uncertain reference ranges [62,63]. Near-term clinical utility is therefore more plausible as trend monitoring or flare detection in defined contexts rather than as a stand-alone diagnostic.

Perfusion and oxygenation monitoring provides another complementary layer [18,63,64]. Near-infrared spectroscopy (NIRS) can non-invasively track tissue oxygenation dynamics during rest and activity and may help interpret endurance limitations or recovery delays when synchronized with EMG and kinematics. While promising, these measures also require standardized protocols and outcome-linked validation to clarify what constitutes clinically meaningful change (e.g., Oswestry Disability Index, pain interference, work ability, activity tolerance).

### 3.5. Integrating Biosensor Data and AI-Driven Interpretation

Here, we focus on transparent, implementation-oriented machine learning (ML) workflows (data quality control, feature engineering, and expert-in-the-loop labeling) rather than proposing a novel algorithm or reporting benchmarked performance metrics. Our emphasis is on long-duration, real-world recordings and on safe model refinement when atypical patterns occur, which are common translational failure points in wearable sensing [65,66]. Multimodal sensing generates heterogeneous, noisy, and context-dependent data. The main opportunity for ML is not “automation for its own sake,” but improving interpretability: identifying robust patterns linked to function, rehabilitation response, and clinically meaningful endpoints when models are trained and evaluated rigorously [67,68,69,70].

In LBP, ML methods have been explored for tasks such as activity recognition, detection of movement strategies (e.g., guarding/co-contraction proxies), within-person monitoring of change, and prediction of response trajectories—typically by combining IMU, EMG, and (where available) physiological features [27,55,71,72]. However, generalizability is often constrained by small datasets, inconsistent protocols, and limited external validation. For clinical translation, models should be explainable, trained on well-defined outcomes, and embedded within workflows that support clinician oversight rather than replacing judgment.

An important practical role for human oversight is handling atypical signals and unexpected patterns during real-world deployment [73]. Semi-automatic expert-in-the-loop approaches can support training-set curation, robust labeling, and safer model updates when new patterns emerge (Figure 2). Ultimately, successful integration will depend on: (i) transparent preprocessing and reporting, (ii) reference datasets linking biosensor metrics to validated clinical outcomes, (iii) interoperability standards, and (iv) interfaces that deliver actionable summaries rather than raw signals. Taken together, these elements define an implementation blueprint for clinically usable biosensing analytics rather than a claim of algorithmic novelty.

## 4. Implementation, Regulation, and Market Barriers

For biosensor-based technologies to move from prototypes to everyday LBP care, they must fit clinical reality. Devices should be quick to apply, robust to placement variability, and require minimal calibration, with outputs that are interpretable without specialist technical expertise [74]. Adoption also depends on workflow integration—interoperability with electronic health records, telemedicine platforms, and rehabilitation software, automated data quality control, and clinician-friendly dashboards that provide actionable summaries rather than raw streams [75,76,77]. To support clinical governance and safe use, platforms should provide a complete audit trail and model/version control (including parameter changes), ensuring traceability of outputs over time and across care settings. Scalability across settings (tertiary centers, outpatient clinics, and home-based rehabilitation) and low patient burden are equally critical for sustained use.

A major barrier is the evidence burden itself. Beyond technical performance, biosensors must demonstrate measurement validity (that they capture the intended construct), clinical reliability and accuracy under real-world conditions (motion artefacts, missing data, placement variability), and—most importantly—clinical utility (that outputs improve decisions, trajectories, or outcomes) [28]. This requires evaluation that extends from bench and laboratory studies to pragmatic clinical validation and implementation evidence (feasibility, adherence, workflow fit, and adoption), because real-world uptake often becomes the decisive test of translational value.

Implementation is also constrained by regulation, ethics, and economics. In the EU, wearable devices and software used for clinical decision support may fall under medical device regulation, and software components must align with applicable requirements under the EU MDR; AI-enabled systems additionally need to comply with emerging EU AI governance frameworks where relevant [78,79,80]. Data privacy, cybersecurity, and transparent model updating are essential, particularly for cloud-connected and remote-monitoring solutions. Finally, reimbursement and procurement models must recognize the added clinical work and infrastructure, and cost-effectiveness evidence will be needed to justify investment.

Global market momentum is driven by the high prevalence of LBP, capacity constraints in rehabilitation services, and the rapid expansion of tele-rehabilitation and home-based care [1,2,81,82,83]. However, adoption at scale will depend on demonstrating real-world effectiveness and value, and on meeting regulatory and interoperability requirements across health systems.

Despite rapid advances in biosensor hardware and analytics, clinical uptake remains limited and uneven (Table 4). Most solutions still fall short of routine requirements—validated endpoints, robust performance in real-world use, and workflow integration—so patient benefit is currently constrained to selected settings. Closing this implementation gap, rather than further incremental sensing capability, is likely the main determinant of near-term impact.

## 5. Future Developments

**Figure 5:**
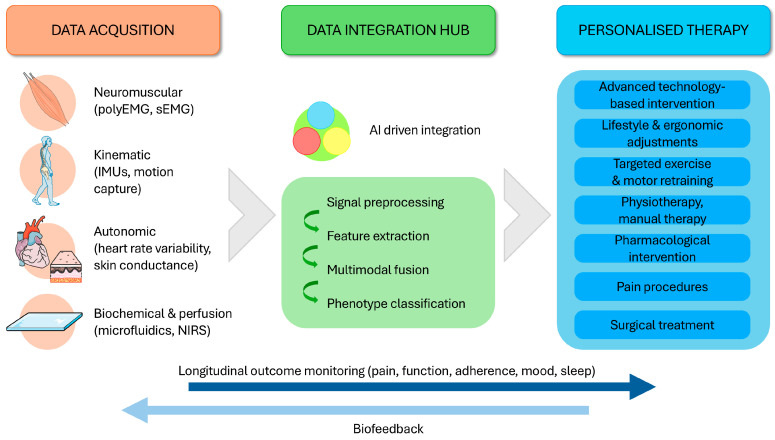
Integration of multimodal biosignals into an adaptive framework for precision, value-based, and patient-centered care. Conceptual model illustrating how multimodal biosignals—neuromuscular, kinematic, autonomic, and biochemical—are integrated within an AI-driven analytical core to identify individual phenotypes and guide personalized treatment strategies. Continuous monitoring of clinical and functional outcomes enables dynamic adjustment of exercise, behavioral, or surgical interventions. Over time, machine-learning analysis of accumulated longitudinal data supports value-based decision-making, ensuring care that is adaptive, data-informed, and centered on the patient’s evolving needs.

The integration of biosensing into LBP care is moving from isolated measurements toward longitudinal, clinically interpretable monitoring that can support more consistent and value-based management (Figure 5). Rather than replacing clinical judgment, the primary goal is to quantify objective correlates of function and recovery—how patients move, load, and adapt in real-world contexts—and to translate these signals into mechanism-informed decisions supported by objective correlates and validated outcomes [56,84,85,86,87].

In the coming years, biosensor-based systems are most likely to add value through treatment stratification within non-surgical care. Multimodal sensing can help characterize rehabilitation-relevant profiles (e.g., guarding/co-contraction-dominant behavior, coordination deficits, fatigue-limited endurance, activity sensitivity) and monitor how these patterns change with therapy [88,89,90]. Combined with patient-reported outcomes, such profiles can support selection and timing of interventions (exercise therapy, education/behavioral strategies, traction where appropriate, and image-guided procedures in selected cases), reducing unwarranted variation and enabling better matching between patient needs and therapeutic options [91,92].

A second near-term contribution is trajectory monitoring with early non-response flags [55,74,84,93]. Repeated sensing across daily life and standardized tasks can identify slow progress, deteriorating movement quality, poor adherence, or adverse physiological responses, prompting timely review and therapy adjustment. Importantly, these systems should function as decision support with explicit uncertainty and clear escalation rules, ensuring clinician oversight and preserving patient autonomy.

Looking ahead, the long-term vision is an incremental closed-loop model of rehabilitation. Initial implementations will remain clinician-supervised and will use simple, transparent rules and feedback to guide exercise progression and self-management [85,94,95,96,97]. As larger, outcome-linked datasets become available and governance matures, more advanced analytics may help personalize progression and predict response trajectories—provided models are interpretable, externally validated, and integrated into clinician-friendly workflows [28,98,99].

Finally, sustainable adoption will depend on implementation realities: usability, interoperability with clinical records, reimbursement alignment, and ethical governance (privacy, transparency, fairness, and safe model updates). Progress will therefore require close clinician-engineer collaboration to ensure that sensing systems deliver actionable summaries—not raw signals—and demonstrate added value in pragmatic trials and real-world implementation studies

## 6. Conclusions

Biosensing for LBP is advancing rapidly, but clinical uptake remains limited and uneven. Most patients cannot yet benefit in routine care because key translational requirements—outcome-linked validation, robust real-world performance, standardized reporting, and workflow/reimbursement integration—lag behind technical capability.

In the near term, the most credible impact lies in clinician-supervised decision support: treatment stratification within non-surgical care, trajectory monitoring, and early non-response flags, integrated with validated self-report and clinical assessment. Overall, closing the implementation gap—rather than incremental sensing novelty—will determine whether biosensing and intelligent analytics deliver scalable, patient-relevant benefit.

## Figures and Tables

**Table 1:** Selected biological, biomechanical, and psychosocial contributors to LBP [1,2,5,6].

**Biological & biomechanical domain**	**Structural abnormalities:** disc degeneration, facet arthropathy, ligamentous and fascial pathology. **Motion impairments:** reduced range of motion, altered kinematics/kinetics and load distribution. **Muscular dysfunction:** delayed activation, asymmetric recruitment, reduced endurance, and motor control deficits. **Neuroimmune–inflammatory processes:** cytokine signaling, local tissue hypoxia, alarmins and related mediators.
**Neurophysiological domain**	**Dorsal root ganglia changes:** structural, functional, and regulatory alterations associated with abnormal spinal loading and sensitization. **Abnormal brain activity:** central sensitization, maladaptive neuroplasticity, altered pain modulation, and pain-related cognitive–affective processing.
**Psychosocial domain**	**Psychosocial influences:** fear-avoidance, stress, loneliness, maladaptive beliefs/catastrophizing, and behavioral adaptation.

**Table 2:** Comparison of biosensing modalities in LBP; overall, most modalities remain at proof-of-concept or small clinical study maturity; standardized, outcome-validated, interoperable end-to-end products for routine LBP care are still largely lacking.

Modality (Typical Placement)	Measurement	Output in LBP Context	Main Strengths	Key Limitations	References
**IMU** (trunk, pelvis, thigh)	Acceleration Angular velocity	Spinal ROM, angular velocity, movement variability, and lumbopelvic coordination	Low burden, ambulatory monitoring, scalable to home use	Drift, misalignment, soft tissue artefacts	Hamersma et al. [9], Laird et al. [10]
**sEMG** (paraspinals, abdominal wall)	Muscle electrical activity	Amplitude, timing, co-contraction, asymmetry	Direct view of neuromuscular strategy, sensitive to fatigue	Motion artefacts, electrode shifts, ECG contamination	Sheeran et al. [11], Boucher et al. [12]
**polyEMG** (paraspinals, abdominal wall)	Muscle electrical activity, spatial activation patterns	Spatial maps, motor unit activation, refined co-activation patterns	Comprehensive evaluation of muscular activity, coordination patterns	Critical electrode placement, computational burden	Murillo et al. [13], Varrecchia et al. [14]
**EEG** (head)	Brain electrical activity	Brain activity alteration related to pain	Research-grade correlates of pain, neuroplasticity mapping	Artifact sensitivity, practical burden for long-term monitoring	Wang et al. [15]
**PPG** (wrist, chest)	Heart rate, heart rate variability, pulse oximetry	Heart rate variability, recovery trends, stress reactivity during activity	Contextualizes stress, autonomic system reaction and recovery	Motion artefacts, sensor placement	Bandeira et al. [16], Espejo-Antúnez et al. [17]
**NIRS** (paraspinals)	Muscle oxygenation	Altered blood flow and muscle oxygenation	Quantification of muscle function and oxygenation	Standardization/ depth/motion artefacts	Lagenfeld et al. [18]
**EDA** (trunk, paraspinals)	Skin conductance	Sympathetic arousal/ stress reactivity	Contextualizes stress, autonomic system reaction to pain	Susceptible to temperature and sweat	van der Miesen et al. [19]

ECG—electrocardiography; EDA—electrodermal activity; EEG—electroencephalography; IMU—inertial measurement unit; NIRS—near-infrared spectroscopy; polyEMG—multi-channel electromyography; PPG—photoplethysmography; ROM—range of motion; sEMG—surface electromyography.

**Table 3:** What clinicians expect from biosensing systems in low back pain (LBP).

**Clinical stratification**	identify common LBP patterns such as degenerative/joint-related, instability-related, inflammatory, myofascial, mixed, or complex variants
**Red flags and triage**	embed/trigger standardized screening and clear escalation rules (progressive neurological deficit, cauda equina features, fracture, infection, malignancy, systemic inflammatory disease)
**Symptom-function linkage**	contextualize pain patterns (including radicular vs. referred/pseudoradicular presentations) while requiring clinical correlation
**Chain-aware fu** **n** **ction assessment**	interpret signals in the context of the pelvis–spine–lower-limb kinetic chain (e.g., hip stiffness, pelvic asymmetry, gait compensation), linking symptoms to task-specific function
**Valid, reliable measurement**	demonstrate construct validity and real-world reliability despite motion artefacts, placement variability, and missing data
**Fast, simple, actionable**	minimal setup/calibration, rapid clinician-friendly summaries with uncertainty, and outputs that map to decisions (treatment stratification, progression, early non-response flags)

**Table 4:** SWOT summary of biosensing in low back pain (LBP).

**Strengths**	Quantifies functional behavior longitudinally (trajectories, adherence, activity exposure) Multimodal sensing (IMU + EMG + physiology) supports triangulation and richer context than single-sensor approaches Enables clinically plausible decision support: treatment stratification and early non-response flags Scalable potential for tele-rehabilitation and home-based monitoring with low patient burden (especially IMU)
**Weaknesses**	Heterogeneous protocols and real-world artefacts (placement variability, motion noise) can bias interpretation Limited outcome-linked validation; many studies are small, heterogeneous, and lack external validation Lack of standards (reporting, ontologies) and limited interoperability with EHR/clinical workflows Interpretation burden and learning curve without clinician-friendly dashboards and uncertainty reporting
**Opportunities**	Pragmatic trials and implementation studies can demonstrate real-world effectiveness and value Standardization deliverables (minimal reporting set, evidence maturity ratings) can accelerate comparability and adoption Incremental closed-loop rehabilitation (rule-based → model-assisted) with clinician oversight Growth of tele-rehab/home care driven by LBP prevalence and capacity constraints
**Threats**	Regulatory and governance requirements (e.g., EU MDR, AI-related compliance) may slow deployment and increase costs Reimbursement and procurement barriers: without cost-effectiveness evidence, routine uptake is unlikely “Hype gap”: overclaims can undermine trust among clinicians, patients, and reviewers Equity risks (digital divide), adherence challenges in older populations, cybersecurity concerns

## Data Availability

Not applicable. No new data were created or analyzed in this study.

## Abbreviations

The following abbreviations are used in this manuscript:

AI	artificial intelligence
CT	computed tomography
EEG	electroencephalography
EMG	electromyography
HRV	heart rate variability
IMU	inertial measurement unit
LBP	low back pain
MDR	Medical Device Regulation
ML	machine learning
MRI	magnetic resonance imaging
NIRS	near-infrared spectroscopy
polyEMG	multi-channel electromyography
sEMG	surface electromyography
